# Regulation of Sphingolipid Metabolism by MicroRNAs: A Potential Approach to Alleviate Atherosclerosis

**DOI:** 10.3390/diseases6030082

**Published:** 2018-09-17

**Authors:** Zainab Jahangir, Ahmed Bakillah, Jahangir Iqbal

**Affiliations:** 1 John P. Stevens High School, North Edison, NJ 08820, USA; 3006676@edison.k12.nj.us; 2 King Abdullah International Medical Research Center, Ministry of National Guard Health Affairs, Al Ahsa 31982, Saudi Arabia; bakillahah@ngha.med.sa

**Keywords:** atherosclerosis, ceramides, lipids, lipoproteins, miRNA, sphingolipids, sphingomyelin

## Abstract

The rapidly expanding field of bioactive lipids is exemplified by the many sphingolipids, which are structurally and functionally diverse molecules with significant physiologic functions. These sphingolipids are main constituents of cellular membranes and have been found associated with plasma lipoproteins, and their concentrations are altered in several metabolic disorders such as atherosclerosis, obesity, and diabetes. Understanding the mechanisms that regulate their biosynthesis and secretion may provide novel information that might be amenable to therapeutic targeting in the treatment of these diseases. Several sphingolipid synthesis genes have been targeted as potential therapeutics for atherosclerosis. In recent years, significant progress has been made in studying the role of microRNAs (miRNAs) in lipid metabolism. However, little effort has been made to investigate their role in sphingolipid metabolism. Sphingolipid biosynthetic pathways involve various enzymes that lead to the formation of several key molecules implicated in atherosclerosis, and the identification of miRNAs that regulate these enzymes could help us to understand these complex pathways better and may prove beneficial in alleviating atherosclerosis.

## 1. Introduction

**Figure 1:**
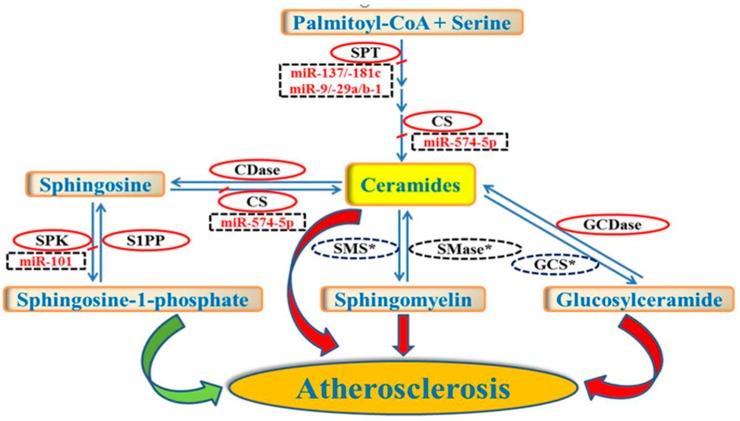
Schematic representation of miRNAs implicated in the synthesis of some key sphingolipid molecules. The figure is representative of some key enzymatic steps involved in sphingolipid biosynthetic pathways that are known to be regulated by various miRNAs (dotted rectangles). Enzymes with asterisks (dotted ovals) may be potential targets for other miRNAs that need to be identified. Increased levels of ceramides, sphingomyelin and glucosylceramide (red arrows) and decreased levels of sphingosine-1-phosphate (green arrow) have been implicated in atherosclerosis. The identification of novel miRNAs that regulate sphingolipid metabolism may be a potential therapeutic target to treat atherosclerosis. Abbreviations: SPT, serine palmitoyl transferase; CDase, ceramidase; CS, ceramide synthase; SPK, sphingosine kinase; S1PP, sphingosine-1-phosphate phosphatase; SMS, sphingomyelin synthase; SMase, sphingomyelinase; GCS, glucosylceramide synthase; GCDase, glucosylceramidase.

High plasma lipid levels are major risk factors for several cardiovascular and metabolic disorders such as atherosclerosis, obesity, and diabetes. Some of the most important risk factors for atherosclerosis are the circulating levels of low-density lipoprotein (LDL) and high-density lipoprotein (HDL) cholesterol [1,2]. Besides traditional risk factors, changes in sphingolipids may contribute to the pathogenesis of cardiovascular disease (CVD) [3,4]. Sphingolipids are a class of lipids that contain a sphingoid base, an aliphatic amino alcohol including sphingosine. Sphingoid bases such as dihydrosphingosine and sphingosine are the fundamental building blocks of all sphingolipids. Sphingolipids are biologically active cell components that regulate cellular processes and play an important role in signal transduction and cellular stress responses. The synthesis and degradation of sphingolipids, which serve as both structural lipids as well as signaling molecules, are regulated to maintain homeostasis [3]. Sphingolipids are either derived from other sphingolipids through catabolism via the salvage pathway or synthesized de novo in the endoplasmic reticulum [5]. Ceramide is a simple sphingolipid composed of sphingosine and a fatty acid. Ceramide constitutes the hydrophobic backbone and serves as the key precursor for the de novo synthesis of other biologically active complex sphingolipids (Figure 1) [6]. It represents a nodal point in the sphingolipid de novo pathway. It can be glycosylated or acquire a polar head group to form glycosphingolipids or sphingomyelin (SM), respectively [3]. It can also be reversibly degraded to form sphingosine which in turn can be phosphorylated by sphingosine kinase (SPK) to form sphingosine-1-phospate (S1P). Once synthesized, sphingolipids can be transported to plasma lipoproteins [7] or remain associated with cellular membranes. An imbalance of sphingolipid levels in plasma and tissue is associated with several metabolic diseases including atherosclerosis [4].

An increased plasma SM level has been proposed as an independent risk factor for coronary heart disease and has been shown to be associated with increased atherosclerosis in humans [8]. A reduction in SM levels in sphingomyelin synthase (SMS) knockout mice is known to decrease atherosclerosis [9,10]. Like SM, increased plasma and aortic ceramide levels are also associated with an increased risk of CVD [11]. Genetic deficiency or the inhibition of type 2-neutral sphingomyelinase (nSMase2), a key enzyme in sphingolipid metabolism, has been shown to decrease atherosclerosis in ApoE knockout mice by reducing inflammatory responses due to a decrease in ceramide levels [12]. Furthermore, plasma glycosphingolipid concentrations have been reported to be elevated in patients at increased risk of atherosclerosis [13]. The pharmacological inhibition of glucosylceramide synthase that is responsible for the synthesis of glucosylceramides has been reported to ameliorate atherosclerosis in ApoE knockout mice and rabbits [14]. In contrast to ceramide, glycosphingolipid, and SM, plasma S1P is believed to be cardioprotective [3]. Regulation of the interconvertible sphingolipid metabolites, ceramide and S1P, and their opposing signaling pathways may determine the net biological effect, a concept referred to as the “sphingolipid rheostat”. Plasma S1P levels significantly decrease after myocardial infarction [15] and increase in patients after percutaneous coronary intervention [16]. It is believed that S1P bound to HDL may predict the severity of coronary heart disease [17]. Recently, it has been shown that apoM acts as a carrier and modulator of S1P that largely affects its homeostasis [18].

Lipid metabolism is a multi-faceted process that involves synthesis, accumulation, secretion, distribution to various tissues, degradation as well as excretion. Lipid metabolism is regulated by finely modulating a set of rate-limiting enzymes and transporters based on the needs of the cells [19]. A variety of cellular regulators including transcription factors that are involved in its synthesis and degradation are responsible for maintaining lipid homeostasis. The discovery that the cellular lipid and lipoprotein metabolism is targeted by miRNAs has provided new insight into the molecular mechanism of the pathogenesis of atherosclerosis, diabetes and obesity [20]. This has led to the exploitation of miRNAs as regulators of lipid metabolism with subsequent potential use in the treatment of lipid metabolism-related disorders [21,22]. This mini-review provides an overview of what is known about the regulation of lipid metabolism in general and sphingolipid metabolism in particular by miRNAs and emphasize their role in atherosclerosis.

## 2. Regulation of Lipid and Lipoprotein Metabolism by miRNAs

miRNAs are a class of small ~22-nucleotide long non-coding sequences which post-transcriptionally regulate gene expression to modulate a wide spectrum of biological processes [23,24]. They act as negative post-transcriptional regulators of gene expression [21,22] and have been proposed to regulate the expression of more than half of the human genes via controlling the expression of mRNA targets. miRNAs repress protein expression by mRNA destabilization and/or translational inhibition after binding the complementary sequences in the 3′ untranslated region (3′-UTR) of target mRNAs via seed sequences (Table 1) [25]. Individual miRNAs can target several mRNAs that are often connected in the same metabolic pathway [26]. Moreover, several miRNAs may regulate a single mRNA, thus enabling a fine-tuning of the targeted mRNA expression, to influence the regulation of cellular events [23,26]. Dysregulation of miRNAs has been correlated with disease pathogenesis [27]. Furthermore, miRNAs can be released from cells as exosomes in many body fluids and may serve as noninvasive biomarkers of diseases [28].

Over the past few decades, the role of miRNAs in controlling the cellular lipid and lipoprotein metabolism has been extensively studied. A large array of miRNAs participate in the lipid and lipoprotein metabolism by targeting enzymes and proteins that are involved in these metabolic pathways. This discovery has provided new insight into the biology and pathophysiology of CVD such as atherosclerosis [20,29]. Higher levels of HDL are inversely correlated with developing CVD. A number of miRNAs, of which miR-33a and miR-33b are the most well-studied, regulate HDL metabolism and control circulating cholesterol levels by regulating HDL synthesis, efflux and clearance [30,31]. miR-33a and miR-33b target multiple genes that regulate cholesterol efflux, which is regarded as an important cholesterol regulatory mechanism [32]. Studies in various animal models and cell lines have confirmed that miR-33 targets ATP-binding cassette (ABC) transporter A1, *ABCA1*, by binding to *3′-UTR* of *ABCA1* via seed sequence to regulate cholesterol metabolism by attenuating circulating HDL levels and thereby decreasing cholesterol efflux to apolipoprotein A1 (apoA1) [30,32]. Conversely, the silencing of miR-33 by using lentiviral expression clone containing anti-sense to miR-33 in experimental animal models generally results in increased hepatic expression of *ABCA1* and enhanced cholesterol efflux to ApoA1, thus increasing circulating HDL levels [32].

In vivo delivery of other miRNAs such as miR-144, miR-758, miR-26, and miR-106b by using either adenoviral or lentiviral expression clones has been shown to have similar results of decreased ABCA1 expression and reduced levels of circulating HDL-cholesterol [33]. The post-transcriptional repression of scavenger receptor class B type 1 (SR-B1) by miR-27, miR-185, miR-96, and miR-223 has also been shown to reduce selective HDL-cholesterol uptake [33]. Reverse cholesterol transport decreases cholesterol levels in peripheral macrophages and in atherosclerotic plaques to increase plaque stability and inhibit atherosclerosis progression [34]. These studies show that HDL metabolism provides a potential therapeutic target to treat atherosclerosis by regulating *ABCA1* via miRNAs.

Besides the HDL metabolism, reports have shown that miRNAs such as miR-30c, miR128-1, or miR-148a are also involved in controlling plasma LDL-cholesterol levels by regulating genes involved in cholesterol biosynthesis, very low-density lipoprotein (VLDL) secretion, and hepatic LDL receptor expression [30]. Recently, it was shown that miR-30c targets microsomal triglyceride transfer protein (MTTP) to regulate VLDL biogenesis and modulate lipid substrate availability for VLDL assembly by targeting genes involved in lipid biosynthesis such as lysophosphatidylglycerol acyltransferase 1 (LPGAT1), ELOVL fatty acid elongase 5 (ELOVL5), stAR related lipid transfer domain containing 3 (STARD3), and membrane bound *O*-acyltransferase domain containing 1 (MBOAT1) [35]. Inhibition of miR-122 has been shown to increase fatty acid oxidation and inhibit lipid synthesis genes such as fatty acid synthase (FASN), steroyl-coA desaturase 1 (SCD1), ATP citrate lyase (ACLY) and acetyl-coA carboxylase 2 (ACC2) and thereby reduce the availability of lipid substrates for VLDL biogenesis [36]. Similarly, inhibition of miR-33a and miR-33b expression in the liver is shown to increase fatty acid oxidation by increasing the expression of carnitine *O*-octanoyltransferase (CROT), carnitine palmitoyltransferase 1A (CPT1A) and hydroxyacyl-CoA dehydrogenase trifunctional multienzyme complex subunit beta (HADHB), and decrease its synthesis by reducing the synthesis of FASN, ACLY, sterol regulatory element binding factor 1 (SREBF1) and acetyl-CoA carboxylase alpha (ACACA) to reduce VLDL secretion in the circulation [37]. Furthermore, reports have suggested that increased expression of miR-155 in liver macrophages regulates fatty acid metabolism by directly targeting liver X receptor alpha (LXRα) and thereby reducing hepatic lipid accumulation [38]. These studies indicate that miRNAs exert their regulatory impacts at different levels of lipoprotein biosynthesis.

## 3. Regulation of Sphingolipid Metabolism by miRNAs

Sphingolipids have been recognized to regulate distinct biological functions beyond their role as structural membrane components. Within the past few decades, significant progress has been made toward understanding the role of sphingolipid pathways for atherosclerosis. The role of miRNAs to regulate sphingolipid metabolism has not been widely studied. There are only a few limited studies, done mostly in cancer cell lines, that have investigated the role of miRNAs in the regulation of enzymes involved in sphingolipid biosynthetic pathways (Figure 1) [39,40,41]. Ceramide is a central molecule in sphingolipid metabolism [6] which is generated either through de novo pathway or salvage pathway [5]. Ceramide acts as a signaling molecule, regulating many cellular responses and functions that may be involved in molecular mechanisms of CVD. The generation of ceramide can be significantly enhanced in certain inflammatory conditions such as atherosclerosis [12]. Ceramide is synthesized by a family of six ceramide synthases (CerS), each of which synthesizes ceramide with distinct acyl chain lengths. An alternatively spliced variant of ceramide synthase, CerS1-2, which is responsible for synthesizing C18-ceramide, is a target for miR-574-5p [41]. The knockdown of miR-574-5p expression has been shown to increase C18-ceramide levels in multiple human cancer cell lines [41].

Serine-palmitoyl transferase (SPT), which converts l-serine and palmitoyl-CoA to 3-ketosphinganine, is the first and rate-limiting enzyme of the de novo biosynthetic pathway of ceramide and SM. SPT has been implicated in the pathogenesis of atherosclerosis and its modulation has been suggested to be a novel therapeutic target in atherosclerosis [42]. Several studies have shown that the pharmacological targeting of SPT through the inhibition with myriocin protects from atherosclerosis in ApoE knockout mice [42,43,44,45]. Myriocin has been shown to reduce the levels of not only ceramide but other sphingolipids downstream of ceramide, such as SM, glucosylceramide and S1P as well [42,44,45]. Similar to pharmacological intervention, targeting the enzymes involved in sphingolipid biosynthesis through miRNAs may be a potential therapeutic intervention for alleviating atherosclerosis. Serine-palmitoyl transferase long chain base subunit 1 (SPTLC1) and 2 (SPTLC2) are the subunits of SPT and one study found a negative correlation between the expression levels of miR-137/-181c and SPTLC1 [40]. Transfection of primary rat astrocytes with miR-137 and miR-181c showed a significant suppression of the endogenous SPTLC1 expression and cellular ceramide levels. On the other hand, anti-miR-137 and anti-miR-181c significantly increased the endogenous SPTLC1 expression and cellular ceramide levels. In the same study, a negative correlation was also found between miR-9/-29a/b-1 and SPTLC2 protein expression in sporadic Alzheimer’s disease brains. Again, the transient transfection of primary rat astrocytes with miR-9, miR-29a, and miR-29b-1 significantly suppressed the endogenous SPTLC2 and cellular ceramide levels and their antagomirs significantly enhanced the expression of SPTLC2 and cellular ceramide levels.

Finally, plasma S1P levels have been reported to be lower in CVD patients, suggesting its involvement in the pathogenesis of atherosclerosis [15]. Sphingosine kinase is involved in the conversion of sphingosine to S1P that have distinct intracellular and extracellular functions [46,47]. Exogenously expressed miR-101 has been shown to down-regulate SPK mRNA and protein expression in colorectal cancer cells [39]. The downregulation of SPK has been shown to result in increased ceramide levels in miR-101 expressed cells. On the other hand, the expression of SPK was enhanced by treating HT-29 human colorectal adenocarcinoma cells with the antagomiR-101 that resulted in lower levels of ceramide [39].

## 4. Conclusions

Significant advances have been made in studying the role of miRNAs in lipid metabolism. However, there is a paucity of information on their role in sphingolipid metabolism. Several sphingolipid synthesis genes have been targeted as potential therapeutics for various metabolic disorders, such as atherosclerosis. Sphingomyelin synthase, sphingomyelinase, and glucosylceramide synthase are some of the key enzymes that have been implicated in atherosclerosis [9,10,12,14] (Figure 1). It is likely that studying the panel of miRNAs that regulate sphingolipid metabolism could help us to understand these pathways better, and the modulation of these pathways may prove to be a potential therapeutic strategy. There are only a few studies that have reported the regulation of enzymes involved in sphingolipid biosynthetic pathways by certain miRNAs [39,40,41], and most of these studies are targeted towards ceramide synthesis in various cancer cell lines [39,41]. However, there is a lack of information on whether miRNAs regulate sphingolipid metabolism in vivo and thereby influence atherosclerosis. Based on the genetic deficiency and pharmacological inhibition studies [9,10,12,14,42], we predict that the regulation of some of the key enzymes in sphingolipid biosynthetic pathways by miRNAs may be useful and a potential therapeutic strategy to alleviate atherosclerosis. Therefore, more focused studies are needed to identify and understand the role of various miRNAs in regulating sphingolipid metabolism.

## Figures and Tables

**Table 1:** Predicted seed sequence, target genes and tissues of miRNAs implicated in lipid and sphingolipid metabolism.

miRNAs	Predicted Seed Sequence *	Target Genes	Target Tissues
**miR-33**	GUUACGU	ABCA1, CROT, CPT1A, HADHB, ACLY, SREBF1, ACACA	Liver
**miR-144**	UAUGACA	ABCA1	Liver
**miR-758**	CAGUGUU	ABCA1	Liver
**miR-26**	AUGAACU	ABCA1	Liver
**miR-106b**	CGUGAAA	ABCA1	Liver
**miR-27**	AUUCGAG	SR-B1	Liver
**miR-185**	AGAGAGG	SR-B1	Liver
**miR-96**	CACGGUU	SR-B1, ABCA1	Liver
**miR-223**	UUGACUG	SR-B1	Liver
**miR-30c**	CAAAUG	MTTP, LPGAT1, ELOVL5, STARD3, MBOAT1	Liver
**miR-128-1**	GCCGGGG	LDLR	Liver
**miR-148**	ACGUGAC	LDLR, ABCA1	Liver
**miR-122**	UGUGAGG	FASN, SCD1, ACLY, ACC2	Liver
**miR-155**	CGUAAU	LXRα	Liver
**miR-574**	GUGUGAG	CerS	Multiple human cancer cells
**miR-9**	UGGUUUC	SPTLC1, SPTLC2	Primary astrocytes
**miR-29a**	UUUAGUC	SPTLC1, SPTLC2	Primary astrocytes
**miR-29b-1**	UUUGGUC	SPTLC1, SPTLC2	Primary astrocytes
**miR-101**	CUAUUGA	SPK	Colorectal cancer cells

* Conserved seed sequence for each miRNA was predicted by using TargetScan Human database (http://www.targetscan.org/vert_72/).

## Abbreviations

ABCA1	ATP-binding cassette transporter A1
ACACA	acetyl-CoA carboxylase alpha
ACC2	acetyl-coA carboxylase 2
ACLY	ATP citrate lyase
ApoA1	apolipoprotein A1
CerS	ceramide synthase
CPT1A	carnitine palmitoyltransferase 1A
CROT	carnitine *O*-octanoyltransferase
CVD	cardiovascular disease
ELOVL5	ELOVL fatty acid elongase 5
FASN	fatty acid synthase
HADHB	hydroxyacyl-CoA dehydrogenase trifunctional multienzyme complex subunit beta
HDL	high-density lipoprotein
LDL	low-density lipoprotein
LPGAT1	lysophosphatidylglycerol acyltransferase 1
LXRα	liver X receptor alpha
MBOAT1	membrane bound *O*-acyltransferase domain containing 1
miRNAs	microRNAs
MTTP	microsomal triglyceride transfer protein
SCD1	steroyl-coA desaturase 1
SM	sphingomyelin
SMS	sphingomyelin synthase
S1P	sphingosine-1-phosphate
SPK	sphingosine kinase
SPT	serine-palmitoyl transferase
SPTLC1	serine-palmitoyl transferase long chain base subunit 1
SPTLC2	serine-palmitoyl transferase long chain base subunit 2
SR-B1	scavenger receptor class B type 1
nSMase2	type 2-neutral sphingomyelinase
SREBF1	sterol regulatory element binding factor 1
STARD3	stAR related lipid transfer domain containing 3
3′-UTR	3′ untranslated region
VLDL	very low-density lipoprotein

## References

[1] Glass C.K., Witztum J.L. (2001). Atherosclerosis. The road ahead. Cell.

[2] Lusis A.J. (2000). Atherosclerosis. Nature.

[3] Borodzicz S., Czarzasta K., Kuch M., Cudnoch-Jedrzejewska A. (2015). Sphingolipids in cardiovascular diseases and metabolic disorders. Lipids Health Dis..

[4] Iqbal J., Walsh M.T., Hammad S.M., Hussain M.M. (2017). Sphingolipids and Lipoproteins in Health and Metabolic Disorders. Trends Endocrinol. Metab..

[5] Pewzner-Jung Y., Ben-Dor S., Futerman A.H. (2006). When do Lasses (longevity assurance genes) become CerS (ceramide synthases)? Insights into the regulation of ceramide synthesis. J. Biol. Chem..

[6] Ogretmen B., Hannun Y.A. (2004). Biologically active sphingolipids in cancer pathogenesis and treatment. Nat. Rev. Cancer.

[7] Iqbal J., Walsh M.T., Hammad S.M., Cuchel M., Tarugi P., Hegele R.A., Davidson N.O., Rader D.J., Klein R.L., Hussain M.M. (2015). Microsomal triglyceride transfer protein transfers and determines plasma concentrations of ceramide and sphingomyelin but not glycosylceramide. J. Biol. Chem..

[8] Jiang X.C., Paultre F., Pearson T.A., Reed R.G., Francis C.K., Lin M., Berglund L., Tall A.R. (2000). Plasma sphingomyelin level as a risk factor for coronary artery disease. Arterioscler. Thromb. Vasc. Biol..

[9] Li Z., Fan Y., Liu J., Li Y., Huan C., Bui H.H., Kuo M.S., Park T.S., Cao G., Jiang X.C. (2012). Impact of sphingomyelin synthase 1 deficiency on sphingolipid metabolism and atherosclerosis in mice. Arterioscler. Thromb. Vasc. Biol..

[10] Liu J., Huan C., Chakraborty M., Zhang H., Lu D., Kuo M.S., Cao G., Jiang X.C. (2009). Macrophage sphingomyelin synthase 2 deficiency decreases atherosclerosis in mice. Circ. Res..

[11] Kasumov T., Li L., Li M., Gulshan K., Kirwan J.P., Liu X., Previs S., Willard B., Smith J.D., McCullough A. (2015). Ceramide as a mediator of non-alcoholic Fatty liver disease and associated atherosclerosis. PLoS. ONE.

[12] Lallemand T., Rouahi M., Swiader A., Grazide M.H., Geoffre N., Alayrac P., Recazens E., Coste A., Salvayre R., Negre-Salvayre A. (2018). nSMase2 (Type 2-Neutral Sphingomyelinase) Deficiency or Inhibition by GW4869 Reduces Inflammation and Atherosclerosis in Apoe (−/−) Mice. Arterioscler. Thromb. Vasc. Biol..

[13] Dawson G., Kruski A.W., Scanu A.M. (1976). Distribution of glycosphingolipids in the serum lipoproteins of normal human subjects and patients with hypo- and hyperlipidemias. J. Lipid Res..

[14] Chatterjee S., Bedja D., Mishra S., Amuzie C., Avolio A., Kass D.A., Berkowitz D., Renehan M. (2014). Inhibition of glycosphingolipid synthesis ameliorates atherosclerosis and arterial stiffness in apolipoprotein E-/- mice and rabbits fed a high-fat and -cholesterol diet. Circulation.

[15] Knapp M., Lisowska A., Zabielski P., Musial W., Baranowski M. (2013). Sustained decrease in plasma sphingosine-1-phosphate concentration and its accumulation in blood cells in acute myocardial infarction. Prostag. Other Lipid Mediat..

[16] Egom E.E., Mamas M.A., Chacko S., Stringer S.E., Charlton-Menys V., El-Omar M., Chirico D., Clarke B., Neyses L., Cruickshank J.K. (2013). Serum sphingolipids level as a novel potential marker for early detection of human myocardial ischaemic injury. Front. Physiol..

[17] Sattler K., Lehmann I., Graler M., Brocker-Preuss M., Erbel R., Heusch G., Levkau B. (2014). HDL-bound sphingosine 1-phosphate (S1P) predicts the severity of coronary artery atherosclerosis. Cell. Physiol. Biochem..

[18] Kurano M., Yatomi Y. (2018). Sphingosine 1-Phosphate and Atherosclerosis. J. Atheroscler. Thromb..

[19] Santulli G. (2015). Effects of low-carbohydrate and low-fat diets. Ann. Intern. Med..

[20] Christian P., Su Q. (2014). MicroRNA regulation of mitochondrial and ER stress signaling pathways: Implications for lipoprotein metabolism in metabolic syndrome. Am. J. Physiol. Endocrinol. Metab..

[21] Novak J., Bienertova-Vasku J., Kara T., Novak M. (2014). MicroRNAs involved in the lipid metabolism and their possible implications for atherosclerosis development and treatment. Mediat. Inflamm..

[22] Wronska A., Kurkowska-Jastrzebska I., Santulli G. (2015). Application of microRNAs in diagnosis and treatment of cardiovascular disease. Acta Physiol. (Oxf.).

[23] Bartel D.P. (2009). MicroRNAs: Target recognition and regulatory functions. Cell.

[24] Filipowicz W., Bhattacharyya S.N., Sonenberg N. (2008). Mechanisms of post-transcriptional regulation by microRNAs: Are the answers in sight?. Nat. Rev. Genet..

[25] Wilczynska A., Bushell M. (2015). The complexity of miRNA-mediated repression. Cell Death Differ..

[26] Lim L.P., Lau N.C., Garrett-Engele P., Grimson A., Schelter J.M., Castle J., Bartel D.P., Linsley P.S., Johnson J.M. (2005). Microarray analysis shows that some microRNAs downregulate large numbers of target mRNAs. Nature.

[27] Ha T.Y. (2011). MicroRNAs in Human Diseases: From Cancer to Cardiovascular Disease. Immune Netw..

[28] Scholer N., Langer C., Dohner H., Buske C., Kuchenbauer F. (2010). Serum microRNAs as a novel class of biomarkers: A comprehensive review of the literature. Exp. Hematol..

[29] Olson E.N. (2014). MicroRNAs as therapeutic targets and biomarkers of cardiovascular disease. Sci. Transl. Med..

[30] Aryal B., Singh A.K., Rotllan N., Price N., Fernandez-Hernando C. (2017). MicroRNAs and lipid metabolism. Curr. Opin. Lipidol..

[31] Zaiou M., Bakillah A. (2018). Epigenetic Regulation of ATP-Binding Cassette Protein A1 (ABCA1) Gene Expression: A New Era to Alleviate Atherosclerotic Cardiovascular Disease. Diseases.

[32] Rayner K.J., Suarez Y., Davalos A., Parathath S., Fitzgerald M.L., Tamehiro N., Fisher E.A., Moore K.J., Fernandez-Hernando C. (2010). MiR-33 contributes to the regulation of cholesterol homeostasis. Science.

[33] Sud N., Taher J., Su Q. (2015). MicroRNAs and Noncoding RNAs in Hepatic Lipid and Lipoprotein Metabolism: Potential Therapeutic Targets of Metabolic Disorders. Drug Dev. Res..

[34] Novak J., Olejnickova V., Tkacova N., Santulli G. (2015). Mechanistic Role of MicroRNAs in Coupling Lipid Metabolism and Atherosclerosis. Adv. Exp. Med. Biol..

[35] Soh J., Iqbal J., Queiroz J., Fernandez-Hernando C., Hussain M.M. (2013). MicroRNA-30c reduces hyperlipidemia and atherosclerosis in mice by decreasing lipid synthesis and lipoprotein secretion. Nat. Med..

[36] Esau C., Davis S., Murray S.F., Yu X.X., Pandey S.K., Pear M., Watts L., Booten S.L., Graham M., McKay R. (2006). miR-122 regulation of lipid metabolism revealed by in vivo antisense targeting. Cell Metab..

[37] Rayner K.J., Esau C.C., Hussain F.N., McDaniel A.L., Marshall S.M., van Gils J.M., Ray T.D., Sheedy F.J., Goedeke L., Liu X. (2011). Inhibition of miR-33a/b in non-human primates raises plasma HDL and lowers VLDL triglycerides. Nature.

[38] Miller A.M., Gilchrist D.S., Nijjar J., Araldi E., Ramirez C.M., Lavery C.A., Fernandez-Hernando C., McInnes I.B., Kurowska-Stolarska M. (2013). MiR-155 has a protective role in the development of non-alcoholic hepatosteatosis in mice. PLoS ONE.

[39] Chen M.B., Yang L., Lu P.H., Fu X.L., Zhang Y., Zhu Y.Q., Tian Y. (2015). MicroRNA-101 down-regulates sphingosine kinase 1 in colorectal cancer cells. Biochem. Biophys. Res. Commun..

[40] Geekiyanage H., Chan C. (2011). MicroRNA-137/181c regulates serine palmitoyltransferase and in turn amyloid beta, novel targets in sporadic Alzheimer′s disease. J. Neurosci..

[41] Meyers-Needham M., Ponnusamy S., Gencer S., Jiang W., Thomas R.J., Senkal C.E., Ogretmen B. (2012). Concerted functions of HDAC1 and microRNA-574-5p repress alternatively spliced ceramide synthase 1 expression in human cancer cells. EMBO Mol. Med..

[42] Hojjati M.R., Li Z., Zhou H., Tang S., Huan C., Ooi E., Lu S., Jiang X.C. (2005). Effect of myriocin on plasma sphingolipid metabolism and atherosclerosis in apoE-deficient mice. J. Biol. Chem..

[43] Park T.S., Panek R.L., Mueller S.B., Hanselman J.C., Rosebury W.S., Robertson A.W., Kindt E.K., Homan R., Karathanasis S.K., Rekhter M.D. (2004). Inhibition of sphingomyelin synthesis reduces atherogenesis in apolipoprotein E-knockout mice. Circulation.

[44] Glaros E.N., Kim W.S., Wu B.J., Suarna C., Quinn C.M., Rye K.A., Stocker R., Jessup W., Garner B. (2007). Inhibition of atherosclerosis by the serine palmitoyl transferase inhibitor myriocin is associated with reduced plasma glycosphingolipid concentration. Biochem. Pharmacol..

[45] Glaros E.N., Kim W.S., Quinn C.M., Jessup W., Rye K.A., Garner B. (2008). Myriocin slows the progression of established atherosclerotic lesions in apolipoprotein E gene knockout mice. J. Lipid Res..

[46] Shida D., Takabe K., Kapitonov D., Milstien S., Spiegel S. (2008). Targeting SphK1 as a new strategy against cancer. Curr. Drug Targets.

[47] Vadas M., Xia P., McCaughan G., Gamble J. (2008). The role of sphingosine kinase 1 in cancer: Oncogene or non-oncogene addiction?. Biochim. Biophys. Acta.

